# Deep learning-based super-resolution dynamic contrast-enhanced radiomics model for predicting NSMP endometrial cancer

**DOI:** 10.3389/fonc.2026.1722374

**Published:** 2026-07-17

**Authors:** Tingting Cui, Jie Ren, Bei Gu, Yongzhao Qin, Yunlong Yue

**Affiliations:** 1 Department of Medical Imaging, Beijing Shijitan Hospital, Capital Medical University, Beijing, China; 2 Department of Obstetrics and Gynecology, Beijing Shijitan Hospital, Capital Medical University, Beijing, China

**Keywords:** dynamic contrast-enhanced, endometrial carcinoma, magnetic resonance imaging, molecular classification, radiomic

## Abstract

**Objectives:**

To determine the performance of a deep learning-based super-resolution (SR) dynamic contrast-enhanced (DCE) radiomic model in predicting nonspecific molecular profile (NSMP) endometrial cancer (EC).

**Methods:**

This study included 140 surgically confirmed EC patients (76 NSMP-type EC patients and 64 non-NSMP-type EC patients) who were randomly divided into training and testing cohorts. Deep learning-based SR reconstruction techniques were applied to convert original-resolution (OR) DCE images into SR images. Radiomic features were extracted from both the SR and OR images. Logistic regression (LR), support vector machine (SVM), and multilayer perceptron (MLP) algorithms were used to develop SR-DCE and OR-DCE models, respectively. Model performance was evaluated via area under the curve (AUC) analysis and decision curve analysis (DCA) methods.

**Results:**

Among the three algorithms (LR, SVM, and MLP), the diagnostic effectiveness of the SR-DCE model was superior to that of the OR-DCE model (P < 0.05). In the testing set, the AUC values for the SR-DCE model were 0.841 (95% confidence interval (CI): 0.724–0.959), 0.800 (95% CI: 0.664–0.937) and 0.764 (95% CI: 0.618–0.911), whereas those for the OR-DCE model were 0.637 (95% CI: 0.464–0.810), 0.603 (95% CI: 0.422–0.785) and 0.656 (95% CI: 0.495–0.818), respectively. Specifically, for the SR-DCE model, both the LR and SVM algorithms exhibited significantly higher AUC values in the testing set than the MLP algorithm did (LR vs. MLP, P = 0.012; SVM vs. MLP, P = 0.041). The clinical utility of the three algorithms was favorable for the SR-DCE model.

**Conclusions:**

Deep learning-based SR reconstruction technology can increase the diagnostic effectiveness of the DCE radiomics model for NSMP EC, and it could become a noninvasive preoperative predictive tool for NSMP EC.

## 1 Introduction

Endometrial cancer (EC) is a common type of malignant gynecological tumor that significantly affects women’s reproductive function and quality of life (1). As the demand for precise and individualized diagnosis and treatment of EC has increased, the molecular classification of this disease has gradually received attention. Clinical trial results have shown that patients with different molecular classifications of EC benefit from adjuvant therapy to varying degrees (2). In 2022, the European Society for Medical Oncology (ESMO) published Clinical Practice Guidelines stating that molecular classification should be incorporated into risk stratification to guide individualized treatment for patients (3). In accordance with the 2020 edition of the World Health Organization (WHO) classification of gynecological tumors, EC is classified into four molecular subtypes: POLE mutation (POLE mut), mismatch repair deficient (MMRd), nonspecific molecular profile (NSMP), and p53 abnormality (p53 abn) (4). In particular, NSMP EC is the most common subtype, accounting for 39% to 67% of all EC cases, and this subtype is mostly of a low grade and detected at the early stage, with a moderate prognosis (5–7). However, conventional molecular classification detection methods (such as next-generation sequencing) are costly and time consuming, and the confirmation of molecular classification on the basis of endometrial biopsies is challenging because of the high heterogeneity in tumors. Therefore, a cost-effective and noninvasive method for predicting NSMP EC is crucial for guiding the preoperative treatment of patients.

Dynamic contrast-enhanced magnetic resonance imaging (DCE-MRI) can be used to clearly visualize subendometrial enhancement (corresponding to the junctional zone), making it a useful tool for the preoperative assessment of EC, such as myometrial invasion (8–10). However, the spatial resolution of DCE-MRI is insufficient, which may not be conducive to providing more internal information about the tumor. With the development of deep learning technology, super-resolution (SR) reconstruction (a technique that is based on a generative adversarial network) has been demonstrated to enhance the spatial resolution and image quality of medical images (11–13). Moreover, many studies have shown that radiomics based on SR reconstruction can increase the diagnostic and predictive effectiveness of the model (14, 15). However, whether the DCE-MRI radiomics model based on SR reconstruction can increase the efficiency of predicting NSMP EC is unknown.

Therefore, the purpose of this study was to enhance the spatial resolution of DCE-MRI images via SR reconstruction and to establish multiple radiomic models to verify their predictive value for NSMP EC.

## 2 Materials and methods

### 2.1 Study population

**Figure 1:**
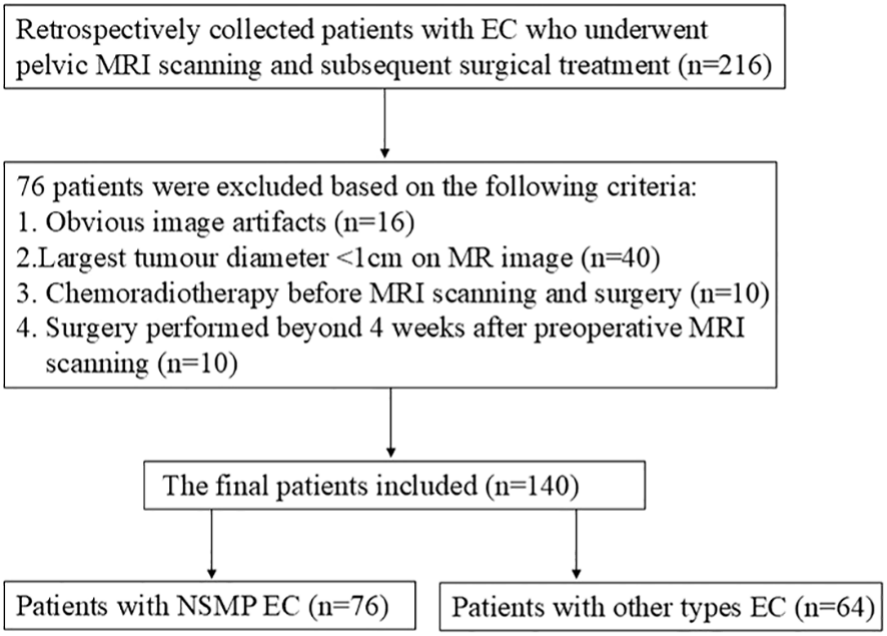
Flowchart of patient recruitment.

This retrospective study was approved by the institutional ethics committee (number: II T 2025-032-001), who waived the need for informed consent. The data included in this study were sourced from patients with EC who underwent pelvic MRI examination and subsequent surgical treatment at our hospital between January 2020 and March 2025. The inclusion and exclusion criteria for patients are shown in **Figure 1**. Finally, 140 patients (76 with NSMP EC and 64 with other types) were included and randomly divided into training and testing cohorts at a ratio of 7:3. Clinicopathological data, including age, tumor grade, myometrial infiltration, lymphovascular space invasion (LVSI), lymph node status and FIGO stage, were collected in baseline form. In this study, tumor staging was based on the 2009 FIGO criteria.

### 2.2 Original DCE-MRI protocol and SR image reconstruction

All pelvic MRI examinations were performed using a 1.5-T MRI scanner (Ingenia, Philips Healthcare, The Netherlands). The original resolution (OR) DCE image acquisition time was 3 min 16 s, and twenty-five dynamic scans were collected with a temporal resolution of 7.8 s. The injection of 0.2 mmol/kg intravenous contrast agent (gadopentetate dimeglumine, CONSUN)) was carried out using a high-pressure syringe. Scanning was performed in the sagittal position, and the detailed acquisition parameters were as follows: repetition time=5.8 ms, echo time=1.73 ms, matrix=188×188, FOV = 300×300 mm^2^, and slice thickness=2.5 mm.

**Figure 2:**
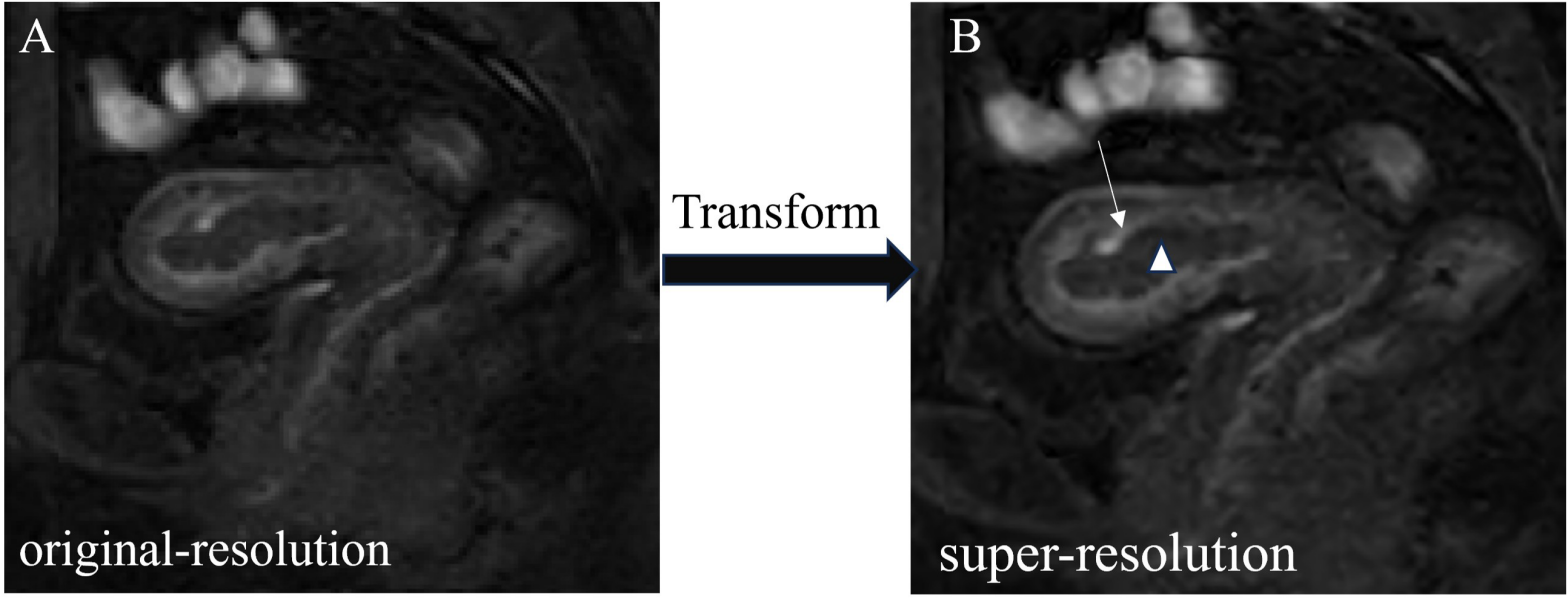
Differences between the OR-DCE image **(A)** and the SR-DCE image **(B)**. The SR-DCE image more clearly shows the enhancement of the tumor (indicated by the triangle) and the tumor–myometrium interface (indicated by the arrow).

In this study, deep learning-based SR reconstruction technology was employed to increase the spatial resolution of DCE images. This technology relies on the original information contained in the images in combination with deep learning algorithms (with a generative adversarial network as the basic architecture) to convert low-resolution images into high-resolution ones, thereby improving the quality of the image. Multiple studies have confirmed that this technology provides excellent stability and reliability and has been widely applied in the field of MRI (16–18). The SR-DCE images in this study were obtained through the Onekey platform (version 3.2; China), and the spatial resolution of these new images was increased fourfold (that is, a pixel volume of 4 × 4 × 4 mm was transformed into 4 × 4 × 1 mm). The newly reconstructed images were named SR-DCE (**Figure 2**).

### 2.3 Image segmentation and radiomic feature extraction

Early DCE images (approximately from the 5th phase) were selected for image segmentation because of their clear visualization of subendometrial enhancement, which corresponds to the tumor contour (10). First, the selected images (including OR and SR images) were subjected to N4 bias field correction to reduce intensity variations caused by magnetic field heterogeneity. These images were subsequently standardized by resampling to 1 × 1 × 1 mm resolution. Tumor contour delineation was performed slice-by-slice by two radiologists (with 7 and 8 years of experience, respectively), who remained blinded to patient information, using ITK-SNAP software (version 4.0). Intra-class correlation coefficient (ICC) was calculated to evaluate the reliability of image segmentation, and radiomic features with ICC > 0.85 were retained.

Radiomic features, including first-order, shape, texture and wavelet features, were extracted separately from the segmented volumes of interest (VOIs) in both the OR and the SR images using PyRadiomics.

### 2.4 Feature selection and model construction

All the features were subjected to Z score standardization to achieve a normal distribution, and the entire feature selection process was conducted solely on the training set data to prevent data leakage from the testing set. First, a t test was applied to evaluate the correlation between features and molecular subtypes, retaining only those with statistically significant associations. Subsequently, Pearson correlation coefficients were calculated to assess inter-feature correlations, with features with coefficients exceeding 0.9 being randomly removed to reduce redundancy. Finally, the least absolute shrinkage and selection operator (LASSO) regression algorithm was used to obtain the optimal feature set. Three machine learning algorithms, namely, logistic regression (LR), support vector machine (SVM), and multilayer perceptron (MLP), were employed to construct radiomic models. To avoid overfitting, we optimized hyperparameters for the models via grid search combined with 5-fold cross-validation on the training set.

### 2.5 Statistical analysis

Continuous variables were described as the mean ± standard deviation (SD), while categorical variables were presented as frequencies and percentages. Differences between NSMP EC and other types were compared using Student’s t test, the Mann–Whitney U test, or the chi-square test. The diagnostic performance of each model was evaluated using receiver operating characteristic (ROC) curves, and the area under the curve (AUC), sensitivity, specificity, accuracy, precision, recall, and F1 score were calculated for both the training and testing sets. Comparisons of AUC values between models were performed using the DeLong test. Decision curve analysis (DCA) was employed to calculate the net benefit of the models across specific threshold ranges, thereby assessing their clinical utility. A P value < 0.05 was considered to indicate statistical significance. Data analysis was conducted using Python (version 3.7.12) and SPSS (version 26.0).

## 3 Results

### 3.1 Clinicopathological characteristics of the participants

The baseline clinicopathological characteristics of the EC patients are detailed in **Table 1**. The results revealed no statistically significant differences between the training and testing sets for any of the evaluated features, except pathological grade in the testing set.

**Table 1:** The baseline clinicopathological characteristics of EC patients^1^.

Characteristic	Training set	Training set	p	Testing set	Testing set	p
	NSMP	non NSMP		NSMP	non NSMP	
Age (years)	54.80 ± 11.34	57.29 ± 10.03	0.253	55.88 ± 14.15	61.31 ± 10.24	0.190
Myometrial invasion			0.287			0.477
<50%	41	34		23	12	
≥50%	9	14		3	4	
Grade			0.240			<0.001
G1	20	13		12	2	
G2	20	19		12	3	
G3	10	16		2	11	
FIGO stage (2009)			0.294			0.234
I	43	38		25	13	
II	4	2		0	0	
III	2	7		1	2	
IV	1	1		0	1	
Lymph node status			0.243			0.271
negative	48	42		26	14	
positive	2	6		0	2	
LVSI			0.050			0.846
negative	42	31		23	13	
positive	2	17		3	3	

FIGO, International Federation of Gynecology and Obstetrics; LVSI, lymphovascular space invasion.

### 3.2 DCE-MRI radiomic feature selection

A total of 234 first-order features, 14 shape features, and 949 texture features were extracted from early DCE images, and their distribution proportions are shown in **Supplementary Figure S1**. The t tests and correlation analysis results revealed 31 features in the OR-DCE group and 41 features in the SR-DCE group. The LASSO algorithm was subsequently employed for optimal feature selection, ultimately retaining 7 features for model development. The details of the least absolute shrinkage and selection operator (LASSO) method and the weight coefficients of the selected features are shown in **Supplementary Figures S2** and **S3**, respectively.

### 3.3 Multiple model construction and evaluation

**Figure 3:**
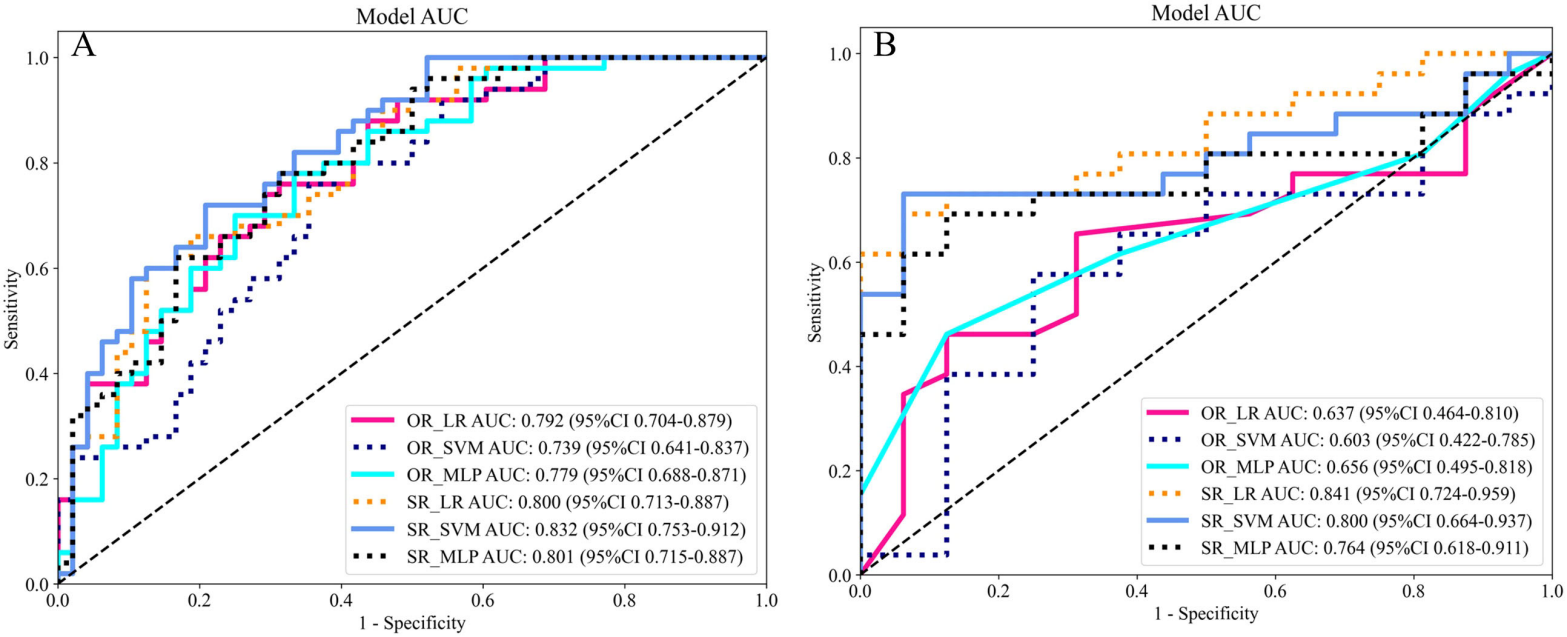
The comparisons of AUC values for OR-DCE and SR-DCE model across the three algorithms. [**(A)** the training set; **(B)** the testing set]. Subfigures **(A)** shows that both models achieve high AUC values across the three algorithms in the training sets; subfigures **(B)** shows the AUC values of the SR-DCE model are higher than those of the OR-DCE model across the three algorithms in the testing set.

**Figure 4:**
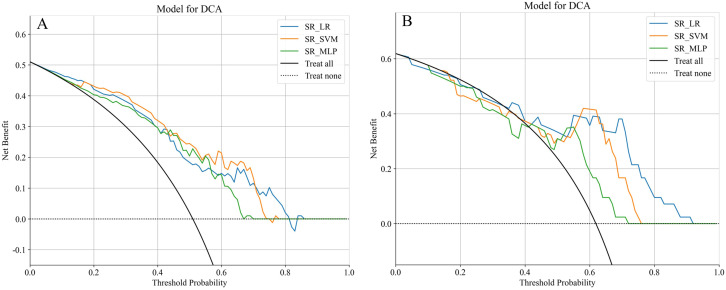
Decision curve analysis for SR-DCE model across the three algorithms. [**(A)** the training set; **(B)** the testing set]. Subfigures **(A, B)** demonstrate that the SR-DCE model yields promising clinical value in both the training and testing sets.

The final retained features were used in combination with the LR, SVM, and MLP algorithms for training and testing to develop predictive models. As shown in **Figure 3** and **Table 2**, in the training set, the AUC values for the SR-DCE model across the three algorithms were 0.800 (95% confidence interval (CI): 0.713–0.887), 0.832 (95% CI: 0.753–0.912) and 0.801 (95% CI: 0.715–0.887), whereas those for the OR-DCE model were 0.792 (95% CI: 0.704–0.879), 0.739 (95% CI: 0.641–0.837) and 0.779 (95% CI: 0.688–0.871), respectively. In the testing set, the AUC values for the SR-DCE model were 0.841 (95% CI: 0.724–0.959), 0.800 (95% CI: 0.664–0.937) and 0.764 (95% CI: 0.618–0.911), whereas those for the OR-DCE model were 0.637 (95% CI: 0.464–0.810), 0.603 (95% CI: 0.422–0.785) and 0.656 (95% CI: 0.495–0.818), respectively. Notably, the AUC values of the SR-DCE model were significantly greater than those of the OR-DCE model across the three algorithms in the testing set (P < 0.05). Specifically, for the SR-DCE model, compared with the MLP algorithm, both the LR and the SVM algorithms resulted in significantly higher AUC values in the testing set (LR vs. MLP, *P* = 0.012; SVM vs. MLP, *P* = 0.041); the values of the LR and the SVM were comparable (P = 0.128). Within a defined threshold range, the clinical utility of the SR-DCE model was favorable across the three algorithms, as shown in **Figure 4**.

**Table 2:** The diagnostic performance of radiomic models for predicting NSMP EC^1^.

Model	Cohort	Accuracy	AUC	95%Cl	Sensitivity	Specificity	PPV	NPV	Precision	Recall	F1 score
OR_LR	Training	0.724	0.792	0.704-0.879	0.740	0.708	0.725	0.723	0.725	0.740	0.733
OR_SVM	Training	0.704	0.739	0.641-0.837	0.760	0.646	0.691	0.721	0.691	0.760	0.724
OR_MLP	Training	0.724	0.779	0.688-0.871	0.700	0.750	0.745	0.706	0.745	0.700	0.722
SR_LR	Training	0.735	0.800	0.713-0.887	0.600	0.875	0.833	0.677	0.833	0.600	0.698
SR_SVM	Training	0.755	0.832	0.753-0.912	0.720	0.792	0.783	0.731	0.783	0.720	0.750
SR_MLP	Training	0.735	0.801	0.715-0.887	0.780	0.687	0.722	0.750	0.722	0.780	0.750
OR_LR	Testing	0.667	0.637	0.464-0.810	0.654	0.687	0.773	0.550	0.773	0.654	0.708
OR_SVM	Testing	0.643	0.603	0.422-0.785	0.577	0.750	0.789	0.522	0.789	0.577	0.667
OR_MLP	Testing	0.619	0.656	0.495-0.818	0.462	0.875	0.857	0.500	0.857	0.462	0.600
SR_LR	Testing	0.786	0.841	0.724-0.959	0.692	0.937	0.947	0.652	0.947	0.692	0.800
SR_SVM	Testing	0.810	0.800	0.664-0.937	0.731	0.937	0.950	0.682	0.950	0.731	0.826
SR_MLP	Testing	0.762	0.764	0.618-0.911	0.692	0.875	0.900	0.636	0.900	0.692	0.783

LR, logistic regression; SVM, support vector machine; MLP, multilayer perceptron; AUC, the area under curve; 95%CI: 95% confidence interval; PPV, positive predict value; NPV, negative predict value.

## 4 Discussion

As the most common molecular subtype, accurate and noninvasive preoperative prediction of NSMP EC can provide evidence for clinicians to assess patient risk stratification, thereby sparing unnecessary lymphadenectomy, radiotherapy and chemotherapy. Although DCE-MRI is a crucial tool for the preoperative assessment of EC, its practical application often prioritizes temporal resolution at the expense of spatial resolution. This compromise may limit the extraction of additional radiomic features associated with tumor heterogeneity. Therefore, adopting deep learning-based SR technology to enhance the image spatial resolution and develop predictive models represents a promising direction for increasing the predictive performance for NSMP EC.

At present, SR reconstruction technology is used for the diagnosis and prognosis prediction of multiple-system lesions. Li C et al. (14) indicated that SR-based radiomic models demonstrate favorable feasibility and effectiveness in predicting uterine fibroid prognosis. Shiraishi K et al. (12) revealed the potential of SR deep learning reconstruction techniques to enhance prostate PI-RADS score assessment capabilities. In this study, we employed 3D deep learning-based SR reconstruction to convert low-resolution pelvic DCE images into SR images with a resolution increase of 4 times. Radiomic models were established using the LR, SVM, and MLP algorithms on the basis of OR-DCE and SR-DCE images to validate their clinical value in predicting NSMP EC. In both the training and testing sets, compared with the OR-DCE models, the SR-DCE models consistently exhibited higher AUC values across all the algorithms, demonstrating significant predictive potential for NSMP EC. These findings provide robust data support for noninvasive preoperative molecular subtyping of EC. For the SR-DCE model, the AUC values among the three algorithms were comparable in the training set. However, in the testing set, compared with the MLP (0.764), the LR and SVM achieved higher AUC values (0.841 and 0.800, respectively). In this study, these three algorithms were employed for model construction, primarily because of their ease of implementation and strong generalization capability and because they have been extensively adopted and validated in numerous prior studies (19–22). However, regarding NSMP EC prediction using the SR-DCE model, it is not obvious which algorithm has more advantages. In the testing set of this study, the diagnostic effectiveness of the LR and SVM algorithms is greater than that of the MLP algorithms, but the LR and SVM algorithms are comparable. This may require external data and a larger sample size to confirm the optimal algorithm.

Yue W et al. (23) employed 12 algorithms combined with multisequence MR images to predict the molecular subtypes of EC. Their results demonstrated that the LR algorithm exhibited superior diagnostic performance, achieving an AUC value of 0.71 for NSMP EC prediction, which was lower than that achieved in our study. In our research, the SR-DCE images exhibited greater clarity and provided a more distinct tumor–myometrium interface than the original images did, which was conducive to accurately delineating the volume of the tumor. In our study, the AUC value of the LR in the SR-DCE model of the testing set is 0.841, which is higher than the 0.639 in the OR-DCE model. Obviously, SR-DCE images provide more information on tumor heterogeneity beyond human visual perception, which may increase the predictive ability of the model. Distinct molecular subtypes of EC reflect varied tumor heterogeneity. The finally selected radiomic features in this study were mainly texture features. These features describe spatial patterns of images, uncover detailed structural information, quantify intratumoral textural distributions, and consequently mirror tumor heterogeneity. Conversely, Zhou J et al. (24) employed LR algorithms incorporating both intratumoral and peritumoral features to predict the molecular subtypes of EC, achieving an impressive AUC value of 0.943 for NSMP EC in their testing set. The superior diagnostic performance of their model suggests the potential clinical utility of peritumoral features in improving prediction accuracy. Future investigations will aim to explore whether SR-derived peritumoral features can further enhance the ability to predict NSMP EC.

Limitations of this study include the following: First, it is a single-center study with a small sample size, and the lack of external/multicenter validation inevitably limits the generalizability of our model. In future work, we plan to collect multicenter data to further validate the performance and robustness of the proposed model. Second, as a retrospective study, potential selection biases may exist. Third, this study did not incorporate peritumoral radiomic features. We will consider integrating multi-regional radiomic features from both intratumoral and peritumoral regions to further improve the predictive performance of the models. Finally, the adoption of SR technology increased the workload of manual image segmentation. Future research could explore automatic segmentation techniques to increase efficiency and reproducibility.

## Data Availability

The original contributions presented in the study are included in the article/**Supplementary Material**. Further inquiries can be directed to the corresponding author.
