# Supplementary material for: Deep learning-based super-resolution dynamic contrast-enhanced radiomics model for predicting NSMP endometrial cancer
Source: Front Oncol. 2026 Jul 17;16:1722374. doi: 10.3389/fonc.2026.1722374 (PMC13423732; doi:10.3389/fonc.2026.1722374)
Supplement: Supplementary file 1 [file Presentation1.pptx]

## Slide 1
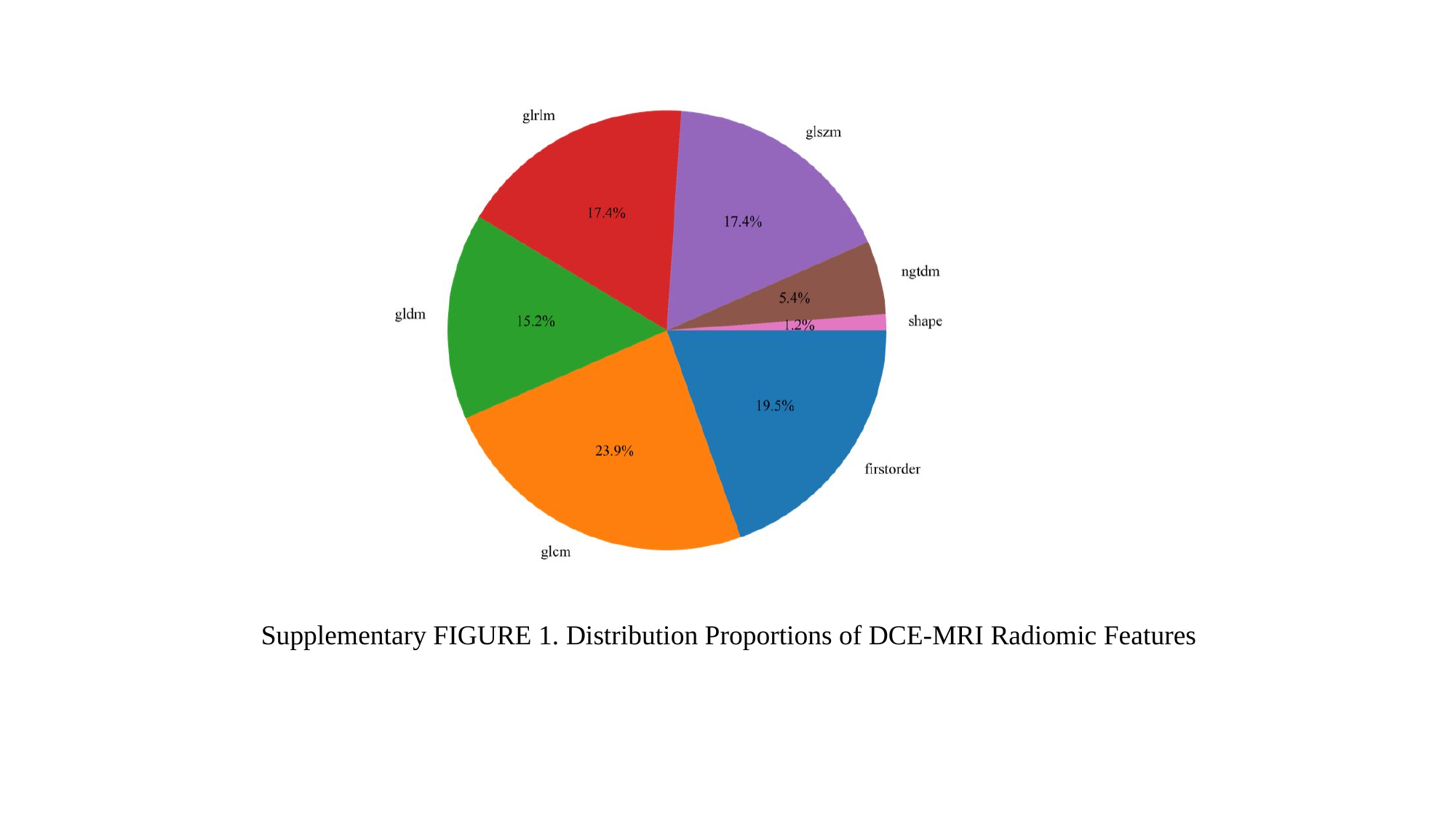

Supplementary FIGURE 1. Distribution Proportions of DCE-MRI Radiomic Features

## Slide 2
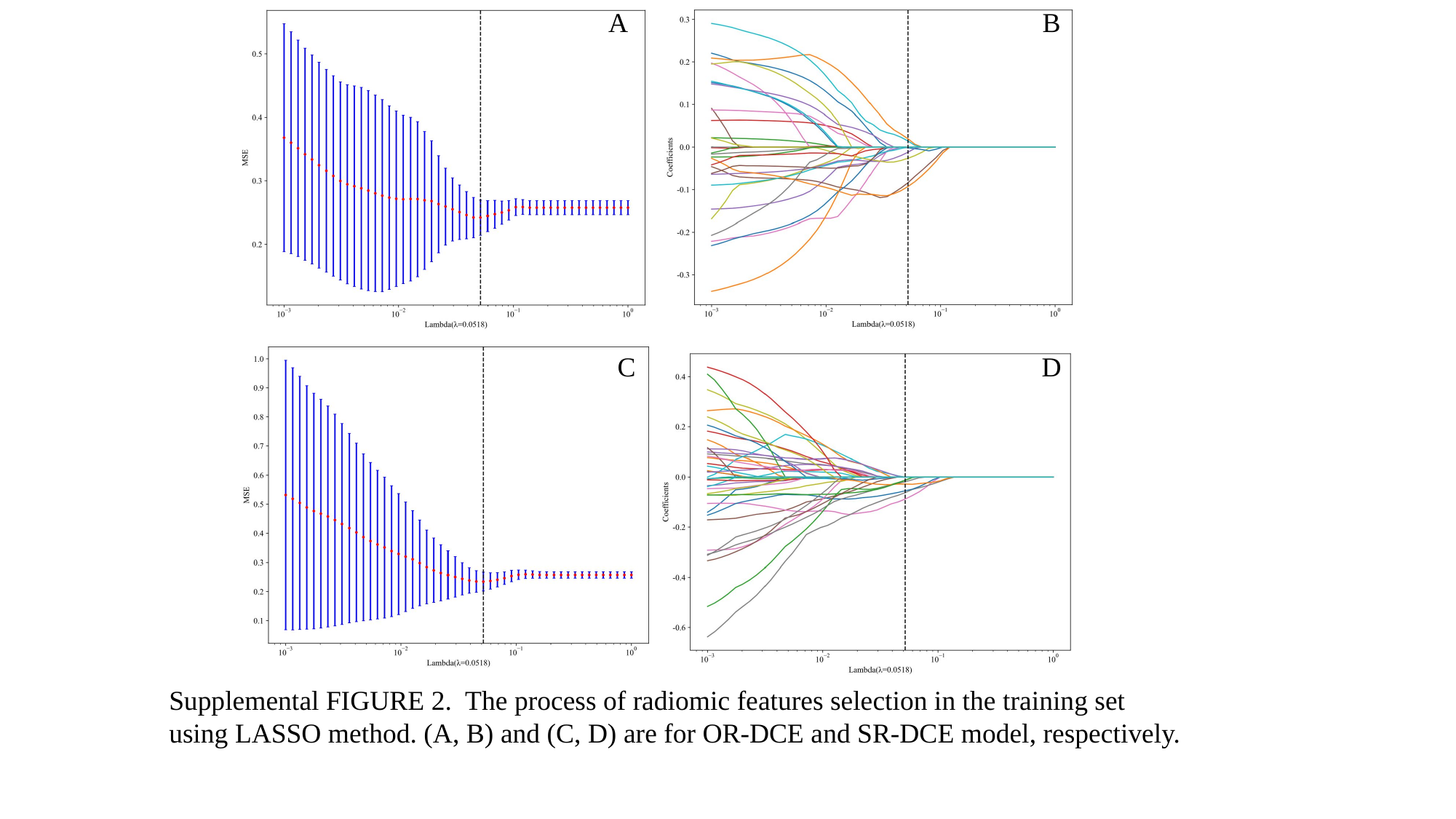

A
B
C
D
Supplemental FIGURE 2. The process of radiomic features selection in the training set
using LASSO method. (A, B) and (C, D) are for OR-DCE and SR-DCE model, respectively.

## Slide 3
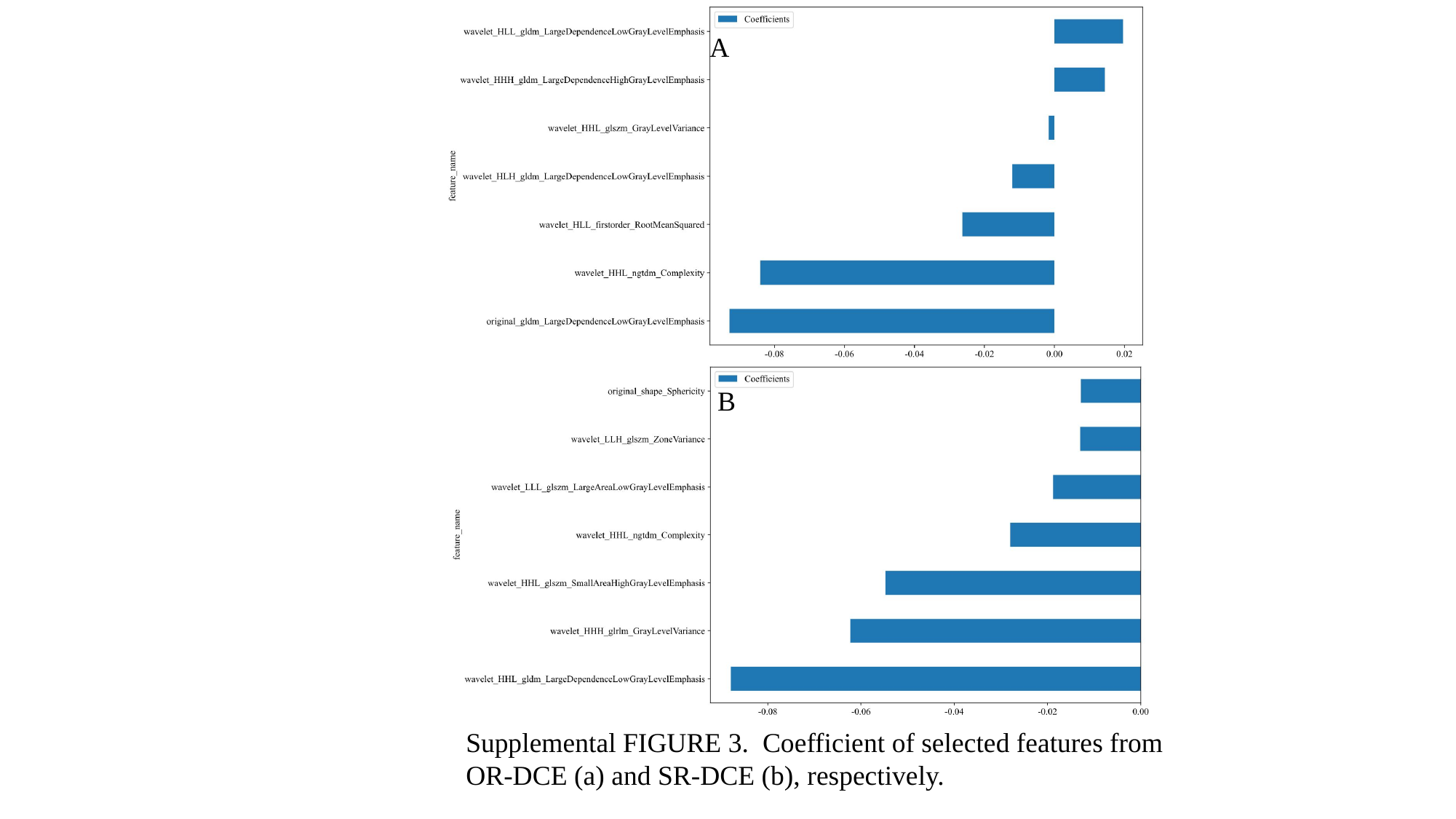

A
B
Supplemental FIGURE 3. Coefficient of selected features from OR-DCE (a) and SR-DCE (b), respectively.
